# Control of Protein and Energy Metabolism in the Pituitary Gland in Response to Three-Week Running Training in Adult Male Mice

**DOI:** 10.3390/cells10040736

**Published:** 2021-03-26

**Authors:** Christina Walz, Julia Brenmoehl, Nares Trakooljul, Antonia Noce, Caroline Caffier, Daniela Ohde, Martina Langhammer, Klaus Wimmers, Siriluck Ponsuksili, Andreas Hoeflich

**Affiliations:** Institute for Genome Biology, Lab Animal Facility, Institute for Genetics and Biometry, Leibniz Institute for Farm Animal Biology (FBN), Wilhelm-Stahl-Allee 2, 18196 Dummerstorf, Germany; walz@fbn-dummerstorf.de (C.W.); trakooljul@fbn-dummerstorf.de (N.T.); noce@fbn-dummerstorf.de (A.N.); caro.caffier@yahoo.de (C.C.); ohde@fbn-dummerstorf.de (D.O.); martina.langhammer@fbn-dummerstorf.de (M.L.); wimmers@fbn-dummerstorf.de (K.W.); ponsuksili@fbn-dummerstorf.de (S.P.)

**Keywords:** pituitary gland, treadmill training, DUhTP mice, pathway analysis, ribosome synthesis, oxidative phosphorylation, miR-124

## Abstract

It is assumed that crosstalk of central and peripheral tissues plays a role in the adaptive response to physical activity and exercise. Here, we wanted to study the effects of training and genetic predisposition in a marathon mouse model on mRNA expression in the pituitary gland. Therefore, we used a mouse model developed by phenotype selection for superior running performance (DUhTP) and non-inbred control mice (DUC). Both mouse lines underwent treadmill training for three weeks or were kept in a sedentary condition. In all groups, total RNA was isolated from the pituitary gland and sequenced. Molecular pathway analysis was performed by ingenuity pathway analysis (IPA). Training induced differential expression of 637 genes (DEGs) in DUC but only 50 DEGs in DUhTP mice. Genetic selection for enhanced running performance strongly affected gene expression in the pituitary gland and identified 1732 DEGs in sedentary DUC versus DUhTP mice. Training appeared to have an even stronger effect on gene expression in both lines and comparatively revealed 3828 DEGs in the pituitary gland. From the list of DEGs in all experimental groups, candidate genes were extracted by comparison with published genomic regions with significant effects on training responses in mice. Bioinformatic modeling revealed induction and coordinated expression of the pathways for ribosome synthesis and oxidative phosphorylation in DUC mice. By contrast, DUhTP mice were resistant to the positive effects of three-week training on protein and energy metabolism in the pituitary gland.

## 1. Introduction

The pituitary gland is located on the lower side of the brain and, under hypothalamic control, regulates growth, reproductive development, stress response, and energy metabolism. Complex endocrine control by the anterior pituitary gland is achieved by the presence of different types and subtypes of cells with distinct gene expression patterns [1,2]. It produces growth hormone (GH), reproductive hormones such as luteinizing hormone (LH) and follicle-stimulating hormone (FSH), adrenocorticotrophic hormone (ACTH), or thyroid-stimulating hormone (TSH), and thus can influence growth and metabolism in multiple tissues. In addition, pituitary glands contribute to energy homeostasis in concert with the hypothalamus, which integrates peripheral and central stimuli in the arcuate nucleus [3]. Due to its central position in hormone production, pituitary glands can also mediate adaptive or even benefitting effects of physical activity. Accordingly, the secretion of growth hormone (GH) is increased in response to resistance training in humans [4]. Notably, exercise induced the secretion of GH to a significantly higher extent than pharmacological stimulation [4]. On the histological level in rats, physical activity induced specific adaptations in somatotropic cells, including increased cell size and the production of larger secretory granules [5]. Exercise in different experimental settings also affected the pituitary–gonadal [6], pituitary–adrenal [7], and pituitary–thyroid [8] hormone axes.

Because the pituitary gland is highly responsive to the effects of physical activity, we postulated that physical activity also induces organ-wide molecular pathways in the pituitary gland not directly related to distinct hormonal axes or distinct cell types. In order to test this hypothesis at the genetic level, we used mice long-term selected for high running performance (DUhTP mice) and unselected controls (DUC) and asked whether we can use bulk RNA-sequencing (RNA-seq) to identify different transcriptional patterns in the pituitary gland in marathon mice and controls. To identify the impact of physical activity, we tested the effects of three-week training in both mouse models. Finally, we asked whether the genetically fixed molecular pathways in pituitary glands of marathon mice (DUhTP) can also be induced by three weeks of training in unselected control mice (DUC). By this approach, we aimed to identify and test transcriptional signatures of physical activity in pituitary glands.

## 2. Materials and Methods

### 2.1. Animals and Study Design

**Figure 1:**
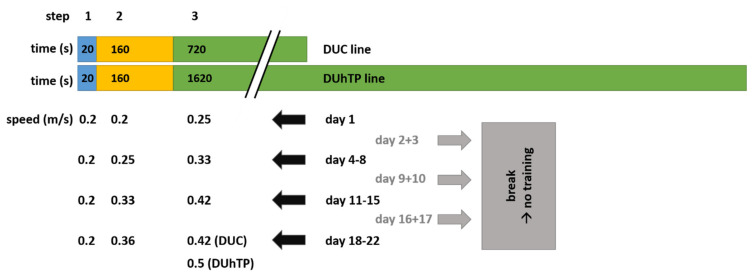
Three-week training program for high running performance (DUhTP) and unselected control (DUC) mice. From day 49, the animals passed a training program on a computer-controlled treadmill for 30 min (DUhTP) and 15 min (DUC) for five days per week, respectively. The duration of running corresponds to 22.56% of their last tested submaximal running performance. The final running speed of a half meter per second was stepwise increased.

All in vivo experiments were performed according to national and international guidelines and were approved by the internal institutional audit committee and by the State of Mecklenburg–Western Pomerania (State Office for Agriculture, Food Safety, and Fisheries; AZ 7221.3-1-014/17, date of approval: 25/04/2017). In this study, we used a long-term selected mouse line (DUhTP), selected for high running endurance, and the corresponding unselected control line DUC [9,10]. The mice were kept under controlled, specified pathogen-free (SPF) conditions in H-Temp Polysulfon cages with a floor area of 370 cm^2^ (Eurostandard Type II, Tecniplast, Hohenpeißenberg, Germany). The animals received fresh drinking water and autoclaved Ssniff^®^ M-Z food (Ssniff-Spezialdiäten GmbH, Soest, Germany) *ad libitum*. Male animals were individually kept in cages from day 21, divided into two groups at 48 days of age, and assigned to a training program (Figure 1; DUC trained (tr.), DUhTP tr.; *n* = 10).

Mice from the sedentary group (*n* = 10) were individually kept in cages for three weeks without any treatment (DUC sedentary (sed.); DUhTP sed.). Both experimental groups from the mouse lines DUC and DUhTP were trained on a treadmill (TSE, Germany) for three weeks. Because DUhTP and DUC mice had different running capacities, DUhTP and DUC were trained for 30 and 15 min per day, respectively. These training intensities correspond to 22.6% of the submaximal treadmill running capabilities in both mouse lines, determined in previous generations ( [11], DUhTP: 2 h 13 min; DUC: 1 h 6 min). At the age of 49 days, the training program started with an initial run by animals of both lines for 15 and 30 min. After a break of 2 days, regular training was started for a total duration of three weeks (5 days training with a break over the weekend). For this purpose, the mice performed a run on a treadmill with an initial speed of 0.2 m/s for 30 m, 0.25 m/s for 50 m, and a final speed of 0.33 m/min. The final distance/speed was increased in weekly intervals up to a final speed of 0.5 m/min (Figure 1). Only the control mice could not manage the final speed of 0.5 m/s, so they completed the last week of training with a final speed of 0.42 m/s. After completing the final run, the mice were sacrificed, and tissue and serum samples were collected. The tissues were weighed, shock-frozen in liquid nitrogen, and stored at −70 °C for subsequent analysis.

### 2.2. Generation of DNA Library

The entire pituitary gland of each animal was homogenized in 1 mL of TRIzol reagent (Invitrogen, Karlsruhe, Germany). Total RNA was extracted in accordance with the manufacturing instructions. The extracted RNA was additionally treated with DNase I using the RNase-free DNase kit (Base-Zero DNase, Biozym, Hessisch Oldendorf, Germany) and with the Zymo^®^ RNA Clean&Concentrator kit (Zymo Research, Irvine, CA, USA). The RNA quality was assessed using an Agilent RNA 6000 Nano kit and 2100 Bioanalyzer (Agilent, Waldbronn, Germany). Samples with RNA integrity numbers (RIN) >8 [12] were used to generate a DNA library using a TruSeq Stranded mRNA Sample Preparation kit in accordance with the manufacture’s protocol (Illumina, Berlin, Germany). Essentially, polyadenylated mRNA molecules were enriched from 2 µg of total RNA using poly-T oligo-coated magnetic beads and chemically fragmented under elevated temperature conditions. The fragmented RNA was then reverse-transcribed into cDNA using random hexamers and Superscript II reverse transcriptase and ligated with TruSeq RNA adapters containing a unique DNA sequencing index to enable multiplexing. The DNA libraries were quality-control assessed for fragment-size distribution using an Agilent Technologies 2100 Bioanalyzer and Agilent DNA-1000 Chip kit.

### 2.3. Next-Generation Sequencing (NGS)

DNA library concentration was quantified using a KAPA qPCR Library Quantification kit (KAPA-Biosystems, Wilmington, MA, USA). Normalized multiplexed DNA libraries with the 0.5% spiked-in PhiX control were clonally cluster amplified using the cBot system and paired-end sequenced for 2 × 101 bp using the high-output mode on a HiSeq2500 (Illumina) at our sequencing facility of the Genome Biology Institute, Leibniz Institute for Farm Animal Biology (FBN), Dummerstorf, Germany.

### 2.4. Differential Gene Expression Analysis

The raw sequencing reads (fastq) were quality-assessed using FastQC (version 0.11.5) (access date: 11/04/2019; http://www.bioinformatics.babraham.ac.uk/projects/fastqc/) and pre-processed by filtering out low-quality reads with a mean Q-score < 30 and read length shorter than 30 bp and trimming adapter-like sequences. High-quality paired-end reads were then aligned to the Ensembl reference mouse genome (GRCm38.p6) using Hisat2 version 2.1.0 [13,14]. The number of reads uniquely mapped to each gene was extracted from the HISAT2 mapping results using HTSeq version 0.8.0 [15].

### 2.5. Data Processing and Evaluation

The resulting gene count data were further analyzed for differently expressed genes (DEGs) using EdgeR [16] and R dependency packages. Genes with lower read values (count per million, cpm) were filtered out to obtain only genes with >0.5 cpm. These had to be present in at least four libraries. EdgeR standard parameters were applied with the trimmed mean of M values (TMM) option considering library size and composition bias and the estimateGLMRobustDisp option to estimate interlibrary variation [16]. The glmFit and glmLRT functions implemented in EdgeR were used for statistical testing of DEGs. The RNA-seq data were then evaluated using the following pipeline: The list contained a cpm value for each analyzed individual and each identified transcript. The average reads cpm values of each transcript were compared between groups in pairs. Line and training comparisons were performed (DUC tr. vs. sed., DUhTP tr. vs. sed., DUhTP sed. vs. DUC sed., DUhTP tr. vs. DUC tr.). The data sets of the four comparison groups then contained a gene identification (Gen-ID), the fold change (log_2_FC), the *p*-value, and the false discovery rate (FDR; Supplementary Table S1). In all analyses, the FDR was defined as the significance level of ≤0.05. For bioinformatic analyses, the DEGs data obtained from the group comparisons were used. For the Venn diagram, the free Venny website (access date: 08/03/2021; https://bioinfogp.cnb.csic.es/tools/venny/) was used [17].

### 2.6. Manhattan Plot

The “ggplot2” R packages [18] were used to perform the mirrored Manhattan plots where only the significant (FDR ≤ 0.05) DEGs in each group comparison were visualized with their genomic position. In these plots, each point represented a gene; the *x*-axis reported the chromosome number, and the *y*-axis showed the DEGs as log_2_FC values. The *y*-axis gray line, referring to the null log_2_FC value, was positioned in the middle of each plot to recognize the upregulated genes as those points shown above that line and the downregulated genes as those points shown under that line.

### 2.7. Validation by Fluidigm

The RNASeq data were validated by 2-step reverse transcription-quantitative PCR (RT-qPCR) using the Fluidigm technique [19]. Thirteen DEGs of isolated pathways were selected to validate if the reads’ cpm was >1 in more than 50% of the samples. Specific primers (Supplementary Table S2) were identified with PrimerBank [20] and blasted using the blastn^®^ software [21] against mouse genome and transcript databases for their suitability in the mouse. mRNA (250–1000 ng) was reverse-transcribed with GOScript™ Reverse Transcriptase kit (Promega). The primers were quality checked with Roche LightCycler^®^ 480 (Roche) using a cDNA dilution of 25 ng to 0.025 ng per reaction. For validation, specific target amplification was performed with the final primer setup and TaqMan PreAmp Mastermix in accordance with the manufacturer’s recommendations. All samples were treated with exonuclease I and diluted. The sample and primer setup followed the instructions for a 48 × 48 array, performing a fast PCR program with a melting curve. Run data were imported and analyzed by data analysis gene (DAG) expression; [22]. For qPCR normalization step, three housekeeping genes were selected within a total of five through the DAG tool Gene Stability Measurement, and the relative expression was calculated for each sample-primer combination. Outliers were identified (Q = 0.1%) and removed using the ROUT method GraphPad Prism V 8.4.2. Group independent correlation analysis was performed for each gene between RNA sequencing signals (cpm) and relative qPCR expression (2^−ΔΔCt^). The Pearson correlation coefficient and *p*-value were calculated and displayed with GraphPad Prism. Group means and log_2_FC calculations were calculated according to RNASeq data.

### 2.8. Pathway Analysis

Ingenuity^®^ Pathway Analysis (IPA^®^, Qiagen, Germantown, MD, USA) was used for the bioinformatic analysis of the holistic data, linking the contents of large data sets from RNA sequencing with the current literature, organizing, linking, and visualizing them [23]. The bioinformatic interpretation was performed with the core analysis of IPA^®^ below the limit of FDR at 0.05. Pathway analysis was performed with the comparison groups and used with the default settings of the program. To indicate significant up- or downregulation pathways, only those with a—log (*p*-value) ≥ 1.3 and a |z-score| ≥ 2 were considered for further interpretation. In addition, we used the freely accessible Pathview website (access date: 08/03/2021; https://pathview.uncc.edu) to visualize KEGG (Kyoto Encyclopedia of Genes and Genomes) paths. The definitions of the options were: for gene data, the DEGs (FDR ≤0.05) loaded with Gen-ID (ENSEMBL) and log_2_FC; as species restricted to *Mus musculus*. The DEGs were manually matched with the KEGG pathways “ribosome” and “oxidative phosphorylation” for all comparison groups.

### 2.9. Localization of DEGs in Published Quantitative Trait Locus (QTLs) Associated with Training Response

Masset and colleagues [24] identified several QTL regions associated with training response in mice after genotyping through a specific single nucleotide polymorphism (SNP) panel [25,26]. We used this information to explore the genome regions around the most important SNPs reported [24] for pre-training, post-training, and change-work in the mouse. We therefore asked if the DEGs identified in the present study were located in the genomic regions described by Masset et al. [24]. To this end, we selected the genome region of 1 megabase (Mb) around the SNPs reported (0.5 Mb upstream and downstream of each SNP). We extracted reference genes present in these regions from the Mouse Genome Database through the search tool “Gene & Markers Query” [27] and compared them with the significant DEGs in each of our group comparisons. The presence of DEGs in genomic regions of previously identified QTLs may add an additional argument for the biomarker content of potential candidate genes identified here.

## 3. Results

### 3.1. RNA-Sequencing Data and Validation by RT-qPCR

Using RNA-seq, an average of 39.3 ± 7.5 million reads per sample were generated in the pituitary gland of DUC mice and 30.7 ± 4.9 million reads per sample in the pituitary gland of DUhTP mice. Around 96% were successfully mapped to the mouse reference genome (GRCm38.p6), and we only selected the mapped reads corresponding to exonic regions, which were approximately 88%. After considering genetic and training comparison, we detected a total of 17,429 expressed genes (data not shown) in the mice’s pituitary gland; 6188 DEGs of them showed a significant (FDR ≥0.05) difference in their expression level between groups (Table 1).

**Figure 2:**
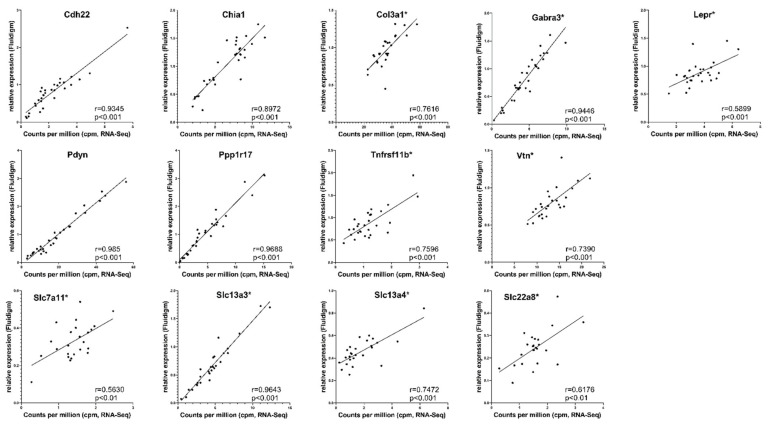
Validation of RNA-seq data by the Fluidigm technique for 13 differentially expressed genes (DEGs) from selected pathways in the pituitary gland. For each gene, the total reads (count per million, cpm) obtained by RNA-seq were plotted on the *x*-axis and RT-qPCR data (2^−ΔΔCt^) on the *y*-axis. Stars at the gene name indicate that outliers were removed during processing, as described in Materials and Methods. Corresponding correlation coefficients (r) and *p*-values are shown.

Single DEGs identified by RNA-seq were cross-validated through Fluidigm assays (Supplementary Table S3). The significant Pearson correlation coefficients ranging from 0.5630 (*p* ≤ 0.01) to 0.9850 (*p* ≤ 0.001) (Figure 2) confirmed a good concordance between RNA-seq and RT-qPCR results for the pituitary gland.

### 3.2. Global Effects of Training and Phenotype Selection on Gene Expression in the Pituitary Gland

As an effect of training, 637 and 50 genes were differentially expressed between the trained and sedentary groups in DUC or DUhTP mice, respectively (Table 1). As an effect of genetic selection, 1732 genes were differentially expressed in sedentary DUhTP versus DUC mice, and 3828 genes were differentially expressed in the trained DUhTP versus DUC mice.

**Figure 3:**
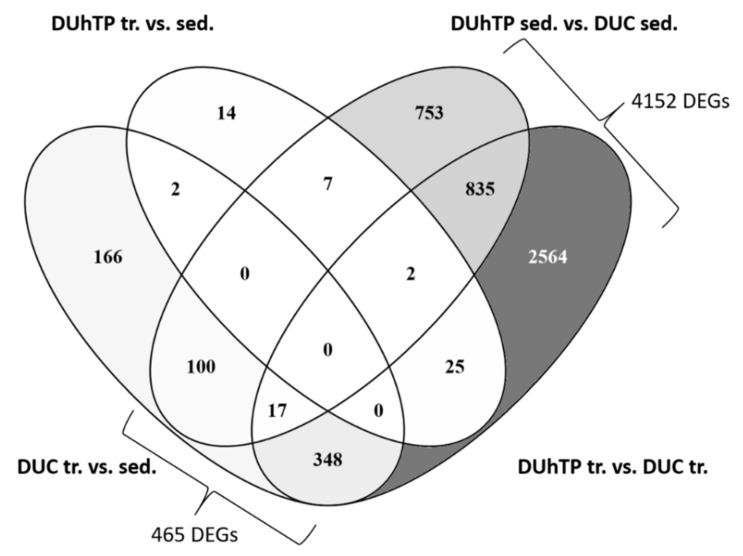
Venn diagram of differentially expressed genes (DEGs) in all comparison groups (FDR ≤ 0.05, sed. = sedentary, tr. = trained, vs. = versus).

The Venn diagram provides the number of common and differently expressed genes in all experimental groups (Figure 3). Training had a clear effect on gene expression in DUC mice’s pituitary glands since 637 transcripts were characterized by altered abundance in response to training. By contrast, in DUhTP mice, only 50 transcripts indicated an effect of training on gene expression in pituitary glands. Notably, from these 50 transcripts, only two transcripts were also regulated in unselected control mice. Furthermore, 465 transcripts regulated by training in DUC mice overlapped with genetic selection for high running performance in untrained and trained DUhTP mice. However, most transcripts identified in DUhTP mice (4152) revealed no overlap with training effects on gene expression identified in unselected control mice. Therefore, the effect of training on gene expression is clear in DUC mice, whereas in DUhTP mice, most of the transcripts affected by training in DUC mice are genetically fixed in DUhTP mice. Almost 90% of the differentially expressed genes in sedentary and trained DUhTP mice versus DUC mice, respectively, had no overlap with the effects observed in trained versus sedentary DUC mice.

### 3.3. Identification of TOP5 Regulated mRNA Transcripts

Table 2 provides the TOP5 up- and downregulated genes in all experimental groups. In response to training, all TOP5 genes differed between the two genetic groups. Accordingly, training had effects on transcripts associated with calcium sensitivity (Syt2, Vsnl1), Mg2+ transport (Cldn19), regulation of post-synaptic actin cytoskeleton modification (Camkv), and glutamate uptake (Grm4) in DUC mice. At the same time, transcripts for ion channels (Kcnj13, Slc6A20, Slc6A12, and Slc22A6) were substantially suppressed in DUC mice in response to training. In DUhTP mice, genes linked with RNA transcription (Rn7sk, Ciart), sodium ion transport (Asic2), blood pressure (Corin), and circulatory regulation (Ciart, Per2) were the most strongly induced ones by training. In addition, training inhibited the abundance of genes associated with oxidative stress (Gstp3) and detoxification (Aldh3b2) as well as receptor binding (Rpsa-ps10, Rxfp1, Cd72) in DUhTP compared to sedentary littermates.

In the Venn diagram, 835 DEGs overlapped in the genetic model (DUhTP vs. DUC sed. and DUhTP vs. DUC tr.). Remarkably, the same TOP5 upregulated genes could be found in both genetic groups (DUhTP vs. DUC sed. and DUhTP vs. DUC tr.). Accordingly, TOP DEGs identified ribosomal proteins 2 and 26, double homoeobox B-like 1, complement component 1 R, and glyceraldehyde-3P-dehydrogenase to be particularly induced in the pituitary gland of DUhTP mice compared to DUC mice. In contrast, transcription for the ribosomal protein L26 and chromobox 3, histone H2a protein type 1, and ATP synthase pseudogene was substantially decreased in trained or sedentary DUhTP mice compared to corresponding unselected controls.

**Figure 4:**
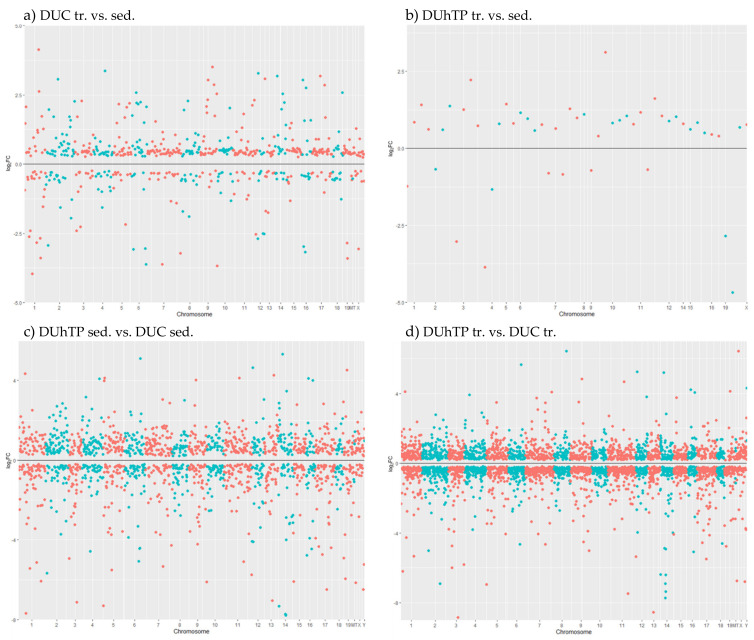
Mirrored Manhattan plots of the significant (FDR ≤ 0.05) differently expressed genes (DEGs) concerning the chromosome location in all comparison groups. Each point represents one gene in its genomic position. The chromosome numbers are reported in the x-axis, the level of expression in the y-axis as log2FC values. The maximum and minimum limits of the y-axis represent the range of log2FC values of each comparison. The gray line in the middle corresponds to log2FC = 0. All points visualized above the line belong to upregulated genes, while all the points visualized under the line are downregulated genes. The comparison groups are shown as (**a**) DUC tr. vs. sed., (**b**) DUhTP tr. vs. sed., (**c**) DUhTP sed. vs. DUC sed., and (**d**) DUhTP tr. vs. DUC tr. Abbreviation: sed. = sedentary, tr. = trained, vs. = versus.

Although several markedly regulated genes (TOP5) were located on chromosome 14 (genetic effect) and chromosome 3 (effect of training; Table 2), the global comparison revealed the contribution of all chromosomes to the differential gene expression in response to phenotype selection or training (Figure 4a–d, Table S1).

### 3.4. Functional Analysis of DEGs

In order to study the potential interaction of genes and pathways related to physical activity and running performance in mice, we compared genes and pathways regulated by both training and phenotype selection. Functional analyses were performed by IPA, including all DEGs, to identify canonical pathways in the experimental groups (Table 3). In response to training, two pathways were activated (EIF2 signaling, oxidative phosphorylation), and four canonical pathways were inhibited (GP6 signaling, cardiogenesis promoting factors, basal cell carcinoma signaling, liver fibrosis signaling) in the pituitary gland from DUC mice. The effect of training on protein metabolism and the oxidative phosphorylation (OXPHOS) pathway was exclusively observed in DUC mice. In fact, training did not affect molecular or metabolic pathways in the pituitary gland of the DUhTP mice at all. In addition, the direct comparison between sedentary DUhTP mice and sedentary DUC mice revealed no effect either on protein metabolism or on the OXPHOS pathway. Instead, two different canonical pathways were significantly inhibited (unfolded protein response and LXR/RXR activation).

However, the comparison of trained DUhTP mice to trained DUC mice demonstrated robust activation of protein metabolism and OXPHOS in trained DUC mice since all, except a few members of both pathways, were significantly inhibited in the pituitary gland of DUhTP animals compared with trained DUC mice. Interestingly, considering genetic selection and running performance enhances the selection-dependent differences between the lines and revealed the significant regulation of additional canonical pathways by training. In trained DUhTP mice, nine canonical pathways were inhibited compared to trained DUC mice. Only the endocannabinoid neuronal synapse pathway was upregulated in trained DUhTP mice vs. trained unselected controls.

By examining genomic regions in the proximity of 11 SNPs previously reported by Masset et al. [24] to be related to training adaptation, we identified which genes changed their expression levels in response to training (DUC tr. vs. sed. and DUhTP tr. vs. sed.), genetic selection (DUhTP sed. vs. DUC sed.), or both together (DUhTP tr. vs. DUC tr.).

The comparisons demonstrated that the selected line activated only one gene (Dnah9) in response to training, which was not present in the unselected line, located on chromosome 11 close to the variant reported for the change-work in Masset et al. [24]. Importantly, no DEGs were found in the selected line in response to activity for pre- or post-training variants, underlining the high performance of this mouse line obtained by long-term genetic selection. In turn, the unselected line activated three genes close to pre-training variants on chromosomes 2 and 8 and one gene close to a post-training variant on chromosomes 14. The Ces1d gene, located on chromosome 8, was found overexpressed in DUhTP mice when comparing both lines in sedentary conditions. Interestingly, in response to training, DUC mice showed an expression increase higher (log_2_FC 0.67) than sedentary DUhTP mice compared to sedentary controls (log_2_FC 0.59). MiR-124a-1hg, identified within the post-training chromosome 14 variants [24], was overexpressed in trained DUC mice compared to sedentary DUC (log_2_FC 2.54) and trained DUhTP mice (log_2_FC 2.03). In DUhTP animals, training did not alter miR124a-1hg expression.

Regarding the response to genetic selection, we found seven genes (including Ces1d mentioned above) distributed on chromosomes 1, 2, 8, and 19 and differentially expressed only in sedentary DUhTP pituitary glands.

Finally, ten DEGs (including Dnah9 and Mir124a-1hg) were found distributed on chromosomes 1, 4, 11, 14, and 19 in response to genetic selection along with training. The genes found on chromosome 2 (Accs) and 19 (Avpi1, Crtac1) were also observed in sedentary DUhTP mice. Table 4 provides information on all DEGs identified in the present study and which are located in the direct neighborhood of known QTLs with an effect on training response in mice [24].

### 3.5. Pathway Analysis

**Figure 5:**
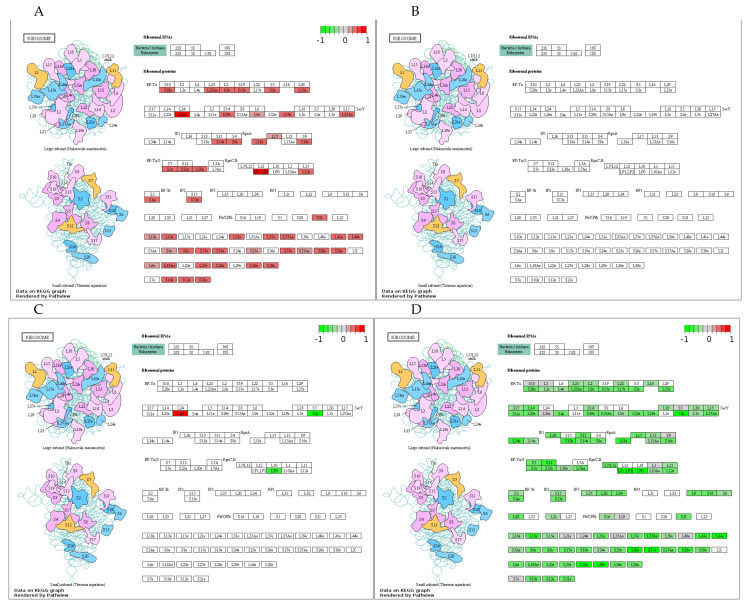
Regulation of ribosome expression in the pituitary gland and the influence of training. KEGG (Kyoto Encyclopedia of Genes and Genomes) pathway analyses of DEGs (FDR ≤ 0.05) in all comparison groups ((**A**): DUC tr. vs. sed.; (**B**): DUhTP tr. vs. sed.; (**C**): DUhTP sed. vs. DUC sed.; (**D**): DUhTP tr. vs. DUC tr.) via https://pathview.uncc.edu (access date: 08/03/2021). Abbreviation: sed. = sedentary, tr. = trained, vs. = versus.

Because the protein metabolic and OXPHOS pathways were consistently elevated in DUC in response to training, both KEGG paths were visualized using Pathview in more detail. Figure 5 shows the training- and line-related effects on gene expression of ribosomal transcripts in the ribosomal pathway called EIF2 signaling. Accordingly, fractions from both the small and large ribosomal subunits were increased by training in DUC mice (Figure 5A). Intriguingly, 49 out of a total of approximately 80 ribosomal proteins were increased, and no inhibitory effect of training on gene expression of the EIF2 pathway was identified in DUC mice. In DUhTP mice, training did not affect gene expression of ribosomal proteins (Figure 5B). Direct comparison of trained DUC and trained DUhTP mice (Figure 5D) revealed a significant increase in RNA transcripts coding ribosomal proteins in the pituitary gland of trained DUC mice compared with trained DUhTP mice, caused by significant transcript upregulation in DUC mice in response to training. Even more, this comparison identified several additional ribosomal proteins regulated by training or by genetic selection for running performance. Accordingly, the present study identified 72 transcripts coding for ribosomal proteins, which are regulated exclusively in control mice in response to training. An effect of training in DUhTP mice was completely absent (Figure 5B).

**Figure 6:**
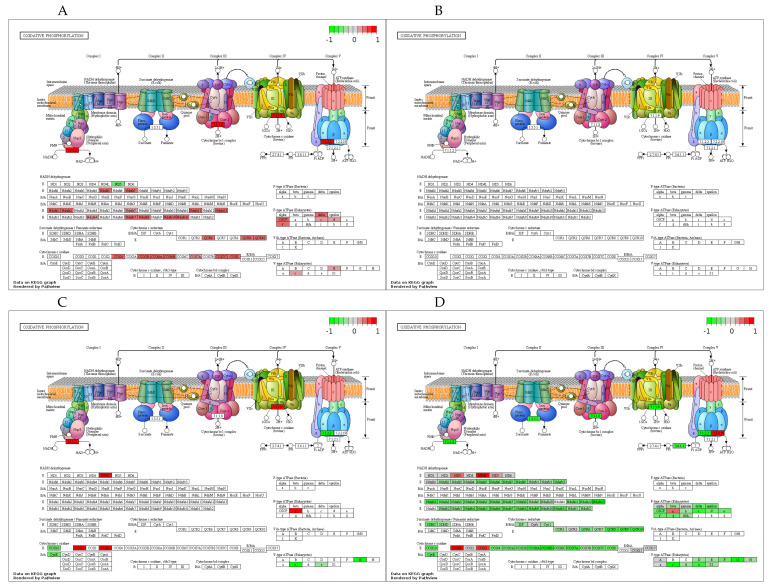
Regulation of oxidative phosphorylation in the pituitary gland influenced by selection and training. The KEGG pathway analyses were obtained by using the DEGs (FDR ≤ 0.05) of all comparison groups ((**A**): DUC tr. vs. sed.; (**B**): DUhTP tr. vs. sed.; (**C**): DUhTP sed. vs. DUC sed.; (**D**): DUhTP tr. vs. DUC tr.) via https://pathview.uncc.edu (access date: 08/03/2021). Abbreviation: sed. = sedentary, tr. = trained, vs. = versus.

As the second canonical pathway significantly activated by training, oxidative phosphorylation was identified in DUC (Figure 6A) but not in DUhTP mice (Figure 6B). In DUC mice, training increased the expression of 34 transcripts coding for OXPHOS proteins, summarized in Figure 6A. Collectively, the activation of these OXPHOS members indicated activation of complexes I, III, IV, and V in DUC mice’s pituitary gland in response to training (Figure 6A). Similar to the protein metabolic pathway, also by comparison of trained DUC vs. trained DUhTP mice, almost all transcripts were significantly higher in unselected controls. Again, several additional transcripts from the OXPHOS pathway were identified by comparing the interaction of training and genetic selection for running performance. Eighty-six transcripts were significantly higher in trained DUC mice than in trained DUhTP mice, and only seven OXPHOS subunits were higher in trained DUhTP vs. trained DUC mice (Table 3 and Figure 6D). Three of the upregulated transcripts belong to the mitochondria-encoded subunits ND3, ND4L, and ND5 of complex I (Figure 6D). Considering the DEGs of the untrained animals of both lines (Figure 6C), a total of 12 OXPHOS transcripts (six up- and six downregulated) were affected. Ten of these transcripts were also regulated by training, whereby two transcripts changed their relative direction of expression. These transcripts were encoding NADH dehydrogenase (7.1.1.2) and cytochrome C oxidase (7.1.1.9).

## 4. Discussion

This study’s original motivation was based on a unique mouse model DUhTP, which has been selected for superior performance for over nearly 35 years [9,10,28]. The advanced running capacities were due to phenotype selection only, and interaction with physical activity was excluded during the selection experiment because running wheels were not offered in the cages. In fact, voluntary physical activity is not elevated in DUhTP compared to unselected controls (DUC) [29]. Because pituitary glands hold a central position in the endocrine control of energy metabolism, we asked whether canonical pathways in this tissue were affected by repeated physical exercise (training) or long-term selection for running performance. In particular, we sought to determine whether phenotype selection for endurance exercise regulates the identical pathways activated by training. In the present study, we addressed tissue-wide adaptations in the pituitary gland and, therefore, studied transcriptome in bulk RNA.

The transcriptome in pituitary glands from phenotype-selected marathon mice (DUhTP) and unselected controls (DUC) was sequenced using an NGS method, and the effects of genotype (DUhTP vs. DUC) and physical activity (trained vs. sedentary) were investigated through comparison of animals from four groups (DUC trained vs. sedentary, DUhTP trained vs. sedentary, DUhTP sedentary vs. DUC sedentary, DUhTP trained vs. DUC trained). Validation by the Fluidigm^®^ technique revealed a significant correlation between RNA sequencing and RT-qPCR results for the tested genes. The total number of pituitary transcripts with more than 17,000 identifications was comparable to 16,654 genes identified before in the same tissue from mice [30], 16,009 genes in cattle breeds [31] but lower than 24,873 genes obtained in laying hens [32]. Notably, on a cellular level, only 4506 different genes were expressed in the pituitary gland from human embryos on average, as demonstrated by single-cell transcriptomics, which is related to the cellular heterogeneity in the pituitary gland and/or due to the developmental status of the tissues [2].

If compared to phenotype selection, training had a moderate effect on gene expression in the pituitary gland. Interestingly, in DUhTP mice, training had almost no effect on gene expression since less than 0.3% of all DEGs were affected by physical activity. Thus, we may conclude that phenotype selection genetically fixed the training-induced metabolic responses in DUhTP mice by direct or indirect mechanisms. In fact, 465 from a total of 637 genes in DUC regulated by training were genetically fixed by phenotype selection in DUhTP mice. This may further imply that although these 465 genes affected by phenotype selection in DUhTP mice have an association with physical activity, the majority of all DEGs in DUhTP vs. DUC mice (*n* = 5560 DEGs) could not directly be linked to physical activity by the present approach. In other words, since the present study may only explain 8.4% of all DEGs in DUhTP mice vs. DUC mice, we may assume that more than 90% of all DEGs are related to longer-term or shorter-term effects or have no function for physical activity.

Nevertheless, the comparison of DEGs in pituitary glands from marathon mice and unselected controls demonstrated that marathon mice are less responsive to training. This may further suggest that specific adaptations in DUC mice in response to training have already resulted from the genetic selection in DUhTP mice. As discussed further down, we may argue that training was perceived on a different level by DUhTP and DUC mice and that endocrine or metabolic feedbacks between peripheral tissues and the pituitary gland were different in both mouse lines. Nevertheless, in DUC mice, the pituitary gland was responsive to the effects of physical exercise.

### 4.1. Genomic Effects and Identification of Candidate Genes

We next addressed the question of whether the DEGs identified by RNA-seq were overrepresented in distinct chromosomes. This question was related to the particular roles postulated for chromosomes 3, 6, 19, and 14 in the adaptive response to exercise in mice [24,33]. Five of the candidate genes present in the TOP5 of the up- and downregulated transcripts in DUhTP vs. DUC mice were located on chromosome 14 (line-associated). Notably, Duxbl1 was the only candidate upregulated in DUhTP vs. DUC mice. Since Duxbl1 is regulated by retinoic acid and involved in the transition of embryonic stem cells to the two-cell state [34], an effect of Duxbl1 on embryonic stem cell reprogramming was discussed. Notably, the retinoic acid pathway was activated in muscle from human subjects in response to resistance training, and thus activation of developmental processes has been debated [35]. Retinoic acids are regulators of growth and development and are required for the adaptive immune response [36]. The retinoic acid pathway was further involved in developmental neurogenesis [37]. Exercise-related neurogenesis was demonstrated in the absence of retinoic acid receptor activation in mice [38]. In addition, chromosomes 3 and 6 were identified by the presence of one DEG per chromosome in DUhTP mice vs. DUC mice. Therefore, we selected the genomic regions close to 11 SNPs reported by Massett et al. (2009) as associated with pre-training, post-training, and work-change in mice. We asked whether DEGs identified by the present study may map to the genomic regions involved in training responses, according to Masset et al. [24]. By this comparison, Dnah9 was identified as a candidate gene in DUhTP mice affected by training. Dnah9 has a role in the proper development of motile monocilia, and loss-of-function mutants are characterized by laterality defects and subtle respiratory ciliary-beating defects in human subjects [39]. A potential effect of Dnah9 for DUhTP mice is not directly evident. Moreover, we identified the Ces1d gene on chromosome 8, overexpressed in sedentary DUhTP and trained DUC mice, suggesting that this gene is genetically fixed in sedentary DUhTP mice related to the selection for high running performance. Ces1d encodes a carboxylesterase in mice and humans and is involved in lipid metabolism [40]. It has been demonstrated that Ces1d has a key role for establishment or maintenance of cytosolic lipid droplets (CLDs) and energy metabolism by regulating lipid transfer rate during development [41,42]. In the context of our study, this would imply that years of selection resulted in increased expression of Ces1d, which is associated with increased formation of cytosolic energy stores in the pituitary gland. As a result of training, Ces1d transcription was further increased in unselected controls, hypothetically leading to increased cytosolic lipid droplets’ formation.

Regarding the variants reported as significant by Masset et al., 2009, we further identified Fhit (fragile histidine triad protein), located close to rs3689508 in chromosome 14, by the comparison of trained DUhTP vs. DUC mice. Fhit represents a tumor repressor [43] and regulates blood pressure in men and mice [44]. Finally, Mir124a-1hg, localized near the SNP rs3660830 on chromosome 14, could be detected with elevated expression in trained DUC but not in trained DUhTP. This marker has a significant effect on post-training effects and training capacity [24]. The gene product, miR-124, plays a role in neurogenesis in the developing [45] and mature brain [46]. In the hippocampus of singly housed mice, exercise reduced miR-124 levels and has been discussed concerning stress resilience [47]. MicroRNAs are further discussed as mediators of beneficial exercise effects on peripheral organs such as the heart [48]. In rats, swimming reduced miR-124 levels and has been discussed in a synergistic relationship with AKT/mTOR signaling, as PI3-K is a target of miR-124 [49]. Therefore, altered miR-124 may have adaptive central and peripheral effects in our experimental system, which opens an interesting field for future studies.

### 4.2. Molecular Pathway Analysis

Long-term selection for high treadmill performance, as a rule, had an inhibitory effect on molecular or metabolic pathways in the pituitary gland. Accordingly, the direct comparison of untrained DUhTP and DUC mice suggested inhibition of the LXR/RXR and the unfolded protein response (UPR) pathway. The UPR pathway is activated in response to cellular stress and is required to control protein quality during protein translation [50]. In the pituitary gland, the UPR pathway was discussed in the context of hormone secretion under the control of selenoprotein T [51]. By contrast, the LXR/RXR pathway seems to play a role in controlling the metabolism and inflammatory response in pituitary glands [52]. In addition, control of the RXR pathway was discussed earlier based on the elevated expression of Duxbl1 in trained DUhTP vs. controls.

In line with the high number of DEGs in trained DUhTP vs. trained DUC mice, several different canonical pathways were affected by the interaction of genetic group and physical activity. The pathway analysis collectively suggested the inhibition of carbon-, lipid-, and protein-metabolism, steroid-signaling, synaptic signaling, and appetite regulation in trained DUhTP vs. trained DUC mice. Integrin signaling was also identified, indicating differential effects on intracellular signaling pathways in trained DUhTP mice vs. unselected controls. Notably, in the liver of untrained DUhTP mice, activation of carbohydrate-, lipid-, and steroid metabolism was described compared to DUC mice [53].

Because EIF2 signaling and the OXPHOS pathways appeared as the only two pathways activated by physical activity, we now discuss potential roles during adaptive response to exercise, respectively. First of all, neither pathway was activated in the pituitary glands of DUhTP mice, suggesting that other adaptations are present that prevent or eliminate activation of both pathways. While this assumption cannot be answered here, we can identify both pathways as the first line of adaptive response in untrained control animals. From approximately 80 ribosomal proteins, 49 members were increased by training in DUC mice. In addition, in response to training, but in human muscle, the EIF2 pathway was identified by IPA [35]. However, a negative effect was found, resulting in the downregulation of 70 rRNA transcripts. In this study, the training period lasted for 20 weeks, resulting in part in a marked hypertrophic response. Accordingly, the different regulation directions could be related to different species, tissues, and training parameters. Accordingly, our study supports regulation of protein synthesis in response to physical activity, initially provided by Phillips et al. [35]. Interestingly, in this study in human muscle, EIF2 signaling was negatively correlated with mTOR pathway activation and lean mass [35]. Ribosome synthesis is a highly coordinated process, and the interaction of different ribosomal proteins establishes two principal functions, namely t-RNA decoding and protein translation [54]. Both functions are separated into two different subunits. Since training had a positive effect on gene expression coding for ribosomal proteins from both subunits, we assumed a coordinated adaptive response in DUC mice resulting in a coordinated increase of both ribosomal subunits resulting in an increased capacity of protein translation in response to training.

Training induced mRNA expression of several oxidative phosphorylation chain subunits, except Mt-Nd5, in unselected controls. Accordingly, gene set enrichment analysis in trained versus untrained control mice identified significant activation of oxidative phosphorylation in pituitary glands. From a significant increase in total oxidative phosphorylation, an increase in energy production could be assumed since this pathway is also associated with pituitary adenoma [55]. Notably, the enhanced energy production could be linked to the elevated demands of protein translation, as discussed earlier. Accordingly, both pathways could be interpreted as a common signature of exercise-related adaptation to increase the pituitary gland’s secretory capacity, whose protective effects could be associated with an improved pituitary function [56].

Notably, the effects of training on both metabolic pathways were not observed in pituitary glands from marathon mice. Instead, trained DUhTP mice were characterized by similar OXPHOS subunits’ expression as untrained controls but by lower expression than trained control mice. This clearly demonstrates that pituitary glands can respond to training by different mechanisms: In untrained DUC mice, molecular pathways of protein synthesis and energy metabolism were increased by training. In DUhTP mice with high endurance exercise performance, these pathways were unaffected by training. Instead, many different transcripts were increased in response to training, suggesting specific effects in different cell populations from the pituitary gland. In addition, the high number of DEGs in untrained DUhTP mice compared to untrained controls may point to a plethora of cell-type-specific adaptations during the long history of phenotype selection in DUhTP mice. Therefore, and as a limitation of the present study, future investigations will have to address these specific effects using single-cell transcriptomics. Nevertheless, the differential response to training in marathon mice and controls could be due to different metabolites in the two mouse lines’ circulation. For example, lactate [57] or butyrate [58] were demonstrated to control GH-secretion by pituitary cells from rats and, therefore, were discussed as potential metabolic effectors of exercise on neuroendocrine responses. As a general limitation, phenotype selection cannot be used for the differentiation of direct versus indirect effects. In order to identify direct or indirect effects on gene expression of the pituitary gland from our animal model, we would have to perform metabolomics studies to identify candidate metabolites, which then could be tested using ex vivo assessment in pituitary explants. However, and as an advantage, results in phenotype-selected models may contain a higher degree of physiological relevance since genotype-based models may not always provide effects that, in fact, play a role under physiological conditions. Finally, non-inbred mice used in the present manuscript have a higher phenotypic variance than inbred mice, which are often used as genotype-based mouse models for functional genome analysis. Accordingly, elevated phenotypic variability could have prevented the detection of hormonal pathways in cellular subpopulations from the pituitary gland. To cope with the issue of sensitivity, other methods like single-cell/nucleus RNA sequencing may be useful, as described earlier in this manuscript. However, since higher phenotypic variance is a function of higher genetic variability due to the non-inbred genetic background present in the animal model used here, it can also be perceived as an advantage due to a better representation of broader populations.

## 5. Summary and Conclusions

An effect of training on the regulation of molecular pathways in the pituitary gland was observed in control mice (DUC) but not in mice long-term selected for high treadmill performance (DUhTP). In particular, two metabolic pathways involved in protein translation and energy metabolism were induced by training in DUC mice. Since both pathways were defined by increased mRNA transcript abundance in response to training and also in comparison to DUhTP mice, we assumed increased protein synthesis and elevated energy supply in DUC mice in response to training. Comparative analysis of DEGs with the literature supported the role of miR-124 for adaptive training responses. Accordingly, miR-124 was decreased by phenotype selection in marathon mice or by exercise in rats [49] and located in the close neighborhood to a QTL region associated with training responses in mice [24]. Accordingly, the role of miR-124, which targets the AKT/mTOR pathway, appears as an interesting subject for subsequent studies in our experimental mouse model.

## Figures and Tables

**Table 1:** Overview of differentially expressed genes (DEGs) in the pituitary glands from trained and sedentary DUhTP and DUC mice with a false discovery rate (FDR) ≤0.05.

Comparison	Number of DEGs (FDR ≤ 0.05)		
	Total	Up	Down
DUC tr. vs. DUC sed.	637	428	209
DUhTP tr. vs. DUhTP sed.	50	38	12
DUhTP sed. vs. DUC sed.	1732	890	842
DUhTP tr. vs. DUC tr.	3828	1617	2211

Numbers of differentially expressed transcripts are shown in black: total genes, in red: upregulated, and in green: downregulated DEGs. Abbreviations: sed. = sedentary, tr. = trained, vs. = versus.

**Table 2:** TOP5 up- and downregulated genes in all comparison groups. The five most upregulated and five most downregulated genes are listed with the corresponding regulation intensity and chromosome localization. Abbreviation: sed. = sedentary, tr. = trained, vs. = versus.

Comparison	Gen-ID	Regulation (log_2_FC)	Description	Chromosome
DUC tr. vs. sed.	Syt2	4.1	Synaptotagmin 2	1
	Camkv	3.5	CaM kinase, vesicle-associated	9
	Cldn19	3.4	Claudin 19	4
	Vsnl1	3.3	Visinin-like 1	12
	Grm4	3.2	Metabotropic glutamate receptor 4	17
	Kcnj13	−4.0	Potassium channel protein	1
	Slc6a20	−3.7	Na^+^- and Cl^-^-dependent transporter	9
	Slc6a12	−3.6	Na^+/^Cl^-^-dependent betaine/GABA transporter	6
	Gm15387	−3.6	High-mobility group 1 protein (pseudogene)	15
	Slc22a6	−3.4	Na^+^-dependent transporter	19
DUhTP tr. vs. sed.	Rn7sk	3.1	snRNA (transcription regulation)	9
	Ciart	2.2	Circadian-associated repressor of transcription	3
	Asic2	1.6	Neuronal sodium channel 1, acid sensitive ion channel 2	11
	Corin	1.4	Serine peptidase, neuronal natriuretic peptide convertase	5
	Per2	1.4	Periodic circadian regulator 2 in neuronal pacemaker	1
	Gstp3	−4.7	Glutathione S-transferase; oxidative stress	19
	Rpsa-ps10	−3.9	Ribosomal protein SA (pseudogene), cell surface receptor	3
	Rxfp1	−3.0	Peptide receptor 1 of the relaxin/insulin-like family	3
	Aldh3b2	−2.9	Aldehyde dehydrogenase	19
	Cd72	−1.3	Lymphocyte receptor	4
DUhTP sed. vs. DUC sed.	Rps2-ps13	6.6	Ribosomal protein2 (pseudogene)	X
	Rps26-ps1	6.2	Ribosomal protein26 (pseudogene)	8
	Duxbl1	5.3	Double homeobox B-like 1	14
	C1r	5.1	Complement component 1 R	6
	Gm4804	4.6	Glyceraldehyd-3P-dehydrogenase (pseudogene)	12
	Gm8104	−8.5	Unknown	14
	Gm7233	−7.7	Unknown	14
	Cbx3-ps7	−7.7	Chromobox 3 (pseudogene)	1
	Gm6356	−7.3	Unknown	14
	Gm15772	−7.3	Ribosomal protein L26 (pseudogene)	5
DUhTP tr. vs. DUC tr.	Rps2-ps13	6.4	Ribosomal protein2 (pseudogene)	X
	Rps26-ps1	6.4	Ribosomal protein26 (pseudogene)	8
	C1r	5.7	Complement component 1 R	6
	Gm4804	5.2	Glyceraldehyd-3P-dehydrogenase (pseudogene)	12
	Duxbl1	5.2	Double homeobox B-like 1	14
	Gm42743	−8.9	Unknown	3
	Hist1h2al	−8.5	Histone H2a-protein type 1, core protein	13
	Gm16440	−7.7	Unknown	14
	Gm10039	−7.5	ATP synthase (pseudogene)	11
	Gm7233	−7.4	Unknown	14

**Table 3:** Significantly, enriched canonical pathways (Fisher’s exact test adjusted *p*-value ≤ 0.05) were deduced from DEGs of the pituitary gland in all comparison groups.

Group	Canonical Pathway	z-Score	Molecules
DUC tr. vs. sed.	EIF2 signaling	4.7	Eif3g, Eif3i, Fau, Hras, Rpl10, Rpl11, Rpl12, Rpl13, Rpl18, Rpl18a, Rpl19, Rpl26, Rpl27, Rpl27a, Rpl28, Rpl30, Rpl31, Rpl35, Rpl36a, Rpl37, Rpl37a, Rpl38, Rpl41, Rpl6, Rpl8, Rpl9, Rplp2, Rps10, Rps12, Rps13, Rps14, Rps15, Rps16, Rps17, Rps19, Rps20, Rps21, Rps23, Rps25, Rps27a, Rps28, Rps29, Rps3, Rps5, Rps6, Rps8, Rps9, Rpsa, Sos2
	Oxidative phosphorylation	4.5	Atp5f1d, Atp5mc2, Atp5mf, Atp5pd, Atp5po, Cox4i1, Cox6a1, Cox6b1, Cox7a2l, Cox8a, Mt-Nd5, Ndufa1, Ndufa11, Ndufa13, Ndufa2, Ndufa7, Ndufb10, Ndufb11, Ndufb4, Ndufb7, Ndufb8, Ndufs7, Uqcr10, Uqcr11
	GP6 signaling pathway	−3.2	Cert1, Col3a1, Col4a1, Col4a2, Col4a3, Col4a4, Col5a1, Lamc3, Prkca, Prkce
	Factors promoting cardiogenesis in vertebrates	−2.5	Bmp6, Bmp7, Camk2a, Crebbp, Prkca, Prkce, Tcf4, Tcf7l2, Tgfbr2, Wnt6
	Basal cell carcinoma signaling	−2.0	Bmp6, Bmp7, Dvl3, Tcf4, Tcf7l2, Wnt6
	Hepatic fibrosis signaling pathway	−2.2	Col3a1, Crebbp, Dvl3, Fth1, Ftl, Hras, Lepr, Pdgfrb, Prkca, Prkce, Rhobtb2, Sos2, Tcf4, Tcf7l2, Tgfbr2, Tnfrsf11b, Wnt6
DUhTP tr. vs. sed.	None		
DUhTP sed. vs. DUC sed.	Unfolded protein response	−2.1	Calr, Cebpg, Cebpz, Dnajc3, Edem1, Hspa1b, Os9, P4hb, Ubxn4
	LXR/RXR activation	−2.5	Abcg4, Alb, Apoa2, Arg2, Ccl2, Cd36, Gc, Hpx, Il1r2, Il33 , Ncor2, Serpina1, Tnfrsf11b, Ttr, Vtn
DUhTP tr. vs. DUC tr.	Oxidative phosphorylation	−7.6	Atp5e, Atp5f1a, Atp5f1b, Atp5f1d, Atp5mc1, Atp5mc2, Atp5mc3, Atp5mf, Atp5mg, Atp5pb, Atp5pd, Atp5pf, Atp5po, Cox10, Cox4i1, Cox5a, Cox6a1, Cox6b1, Cox6c, Cox7a2, Cox7a2l, Cox7b, Cox8a, Cyc1, Cycs, Mt-Co2, Mt-Co3, Mt-Nd4l, Ndufa1, Ndufa10, Ndufa11, Ndufa12, Ndufa13, Ndufa2, Ndufa3, Ndufa4, Ndufa5, Ndufa6, Ndufa7, Ndufa8, Ndufa9, Ndufab1, Ndufb10, Ndufb11, Ndufb2, Ndufb3, Ndufb4, Ndufb5, Ndufb6, Ndufb7, Ndufb8, Ndufb9, Ndufs2, Ndufs3, Ndufs4, Ndufs6, Ndufs7, Ndufs8, Ndufv1, Ndufv2, Ndufv3, Sdhc, Sdhd, Uqcr10, Uqcr11, Uqcrb, Uqcrc1, Uqcrfs1, Uqcrq
	EIF2 signaling	−5.0	Acta1, Akt1, Atf3, Atf4, Atf5, Eif2ak2, Eif2b1, Eif2b3, Eif2b5, Eif3d, Eif3g, Eif3h, Eif3i, Eif3k, Eif3l, Eif4a1, Eif4e, Fau, Hras, Hspa5, Map2k2, Mt-Rnr1, Myc, Pik3cg, Pik3r2, Ppp1ca, Rpl10, Rpl10a, Rpl11, Rpl12, Rpl13, Rpl14, Rpl15, Rpl17, Rpl18, Rpl18a, Rpl19, Rpl23, Rpl27, Rpl27a, Rpl28, Rpl31, Rpl35, Rpl36a, Rpl36al, Rpl37, Rpl37a, Rpl38, Rpl4, Rpl41, Rpl5, Rpl6, Rpl7, Rpl7a, Rpl7l1, Rpl8, Rpl9, Rplp0, Rplp2, Rps10, Rps11, Rps12, Rps13, Rps14, Rps15, Rps15a, Rps16, Rps17, Rps18, Rps19, Rps2, Rps20, Rps21, Rps23, Rps24, Rps25, Rps26, Rps27a, Rps27l, Rps28, Rps29, Rps3, Rps4y1, Rps5, Rps6, Rps8, Rps9, Rpsa
	Synaptogenesis signaling pathway	−2.1	Adcy1, Adcy3, Adcy5, Akt1, Ap1g2, Ap2a1, Ap2a2, Ap2m1, Ap2s1, Arpc2, Arpc3, Arpc4, Arpc5l, Atf2, Atf4, Bad, Bet1l, Calm1 (Includes Others), Camk2b, Cdh1, Cdh12, Cdh24, Cdh6, Cdh8, Cdk5, Cfl1, Clasp2, Cplx3, Creb3, Crebbp, Efna3, Epha6, Gosr2, Grin2c, Grin3a, Grina, Grm4, Hras, Hspa8, Itsn2, Kalrn, Lrrtm2, Map1b, Nap1l1, Nap1l4, Napa, Nlgn2, Nlgn3, Nrxn1, Pik3cg, Pik3r2, Plcg2, Prkag1, Prkar1b, Rab3a, Rab5c, Rac1, Rasgrp1, Reln, Shf, Sncb, Syn3, Syt1, Syt12, Syt14, Syt2, Syt6, Thbs3, Vti1b
	TCA cycle II (eukaryotic)	−2.7	Aco2, Dhtkd1, Fh, Idh3a, Mdh1, Mdh2, Ogdhl, Sdhaf4, Sdhc, Sdhd, Suclg1
	Estrogen receptor signaling	−2.9	Adcy1, Adcy3, Adcy5, Agt, Akt1, Arg2, Atf2, Atf4, Atp5f1a, Atp5f1d, Atp5mc1, Atp5pb, Bad, Cfl1, Creb3, Crebbp, Cyc1, Egfr, Eif2b1, Eif2b3, Eif2b5, Eif4e, Ep300, Esr2, Foxo6, Gna14, Gnal, Gnat2, Gng11, Gng5, Hras, Map2k2, Mdk, Med12, Med21, Mmp14, Mmp17, Mmp19, Mmp20, Mmp7, Myc, Myl12a, Myl6, Myl6b, Myl9, Ncoa1, Ncoa2, Nos3, Pcna, Pdia3, Pgf, Pik3cg, Pik3r2, Plcb2, Plce1, Plcg2, Plch1, Prkaa2, Prkag1, Prkar1b, Prkcg, Sdhc, Sdhd, Shf, Sra1, Thrap3, Uqcrfs1, Vegfd
	Pentose phosphate pathway	−2.4	G6pd, H6pd, Pgd, Rpe, Taldo1, Tkt
	Dolichyl diphosphooligosaccharide biosynthesis	−2.4	Alg1, Alg3, Alg8, Dpagt1, Dpm2, Dpm3
	tRNA charging	−2.1	Farsb, Hars1, Iars2, Kars1, Lars2, Mars1, Nars1, Rars1, Sars1, Tars1, Wars2
	Endocannabinoid neuronal synapse pathway	2.2	Adcy1, Adcy3, Adcy5, Cacna1h, Cacna2d4, Cacnb2, Cacng5, Cacng6, Faah, Gna14, Gnal, Gng11, Gng5, Grin2c, Grin3a, Grina, Kcnj9, Mapk13, Ndufs2, Pdia3, Plcb2, Plce1, Plcg2, Plch1, Prkag1, Prkar1b
	Integrin signaling	−2.1	Acta1, Actn1, Akt1, Arf1, Arf4, Arf5, Arhgap26, Arpc2, Arpc3, Arpc4, Arpc5l, Capn10, Capn5, Capn8, Hras, Itga11, Itga7, Itga8, Itgal, Itgb3, Itgb5, Itgb6, Map2k2, Myl12a, Myl9, Parva, Pfn1, Pik3cg, Pik3r2, Plcg2, Ptk2, Rac1, Rhob, Rhobtb2, Rhof, Rhog, Rnd2, Tnk2, Tspan5, Zyx

Pathways with a |z-score| ≥2 were considered as significantly activated and inhibited in the pituitary of the corresponding group, respectively. Font color of the gene symbol indicates lower expression (green) and higher expression (red) concerning the control for each comparison (unselected or untrained). Abbreviation: sed. = sedentary, tr. = trained, vs. = versus.

**Table 4:** Identification of DEGs, located in the direct neighborhood (0.5 Mb up and downstream) of previously published genetic markers and residing in Quantitative Trait Locus (QTL) regions with significant effects (marked with an asterisk) on training response in mice [24].

			Response to Exercise		Response to Genetic Selection	Response to Genetic Selection with Exercise
Condition	SNPs	Chromosome: Position (bp)	DUC tr. vs. sed.	DUhTP tr. vs. sed.	DUhTP sed. vs. DUC sed.	DUhTP tr. vs. DUC tr.
Pre- training	rs4222922	1: 193,272,691	NA	NA	Traf3ip3	Gm37691, Irf6, Hsd11b1, Syt14
	rs4223268	2: 93,419,862	Alx4, Tspan18	NA	Accs	Accs
	rs368717 *	3: 120,768,827	NA	NA	NA	NA
	rs3089148	8: 92,941,850	Ces1d	NA	Ces1d	NA
	rs3689508 *	14: 9,760,330	NA	NA	NA	Fhit
	rs3679049 *	19: 42,060,307	NA	NA	Avpi1, Crtac1, Marveld1	Avpi1, Crtac1, Exosc1, Rrp12
Post- training	rs368717 *	3: 120,768,827	NA	NA	NA	NA
	rs3667625	4: 54,194,913	NA	NA	NA	Rps15a-ps8
	rs3660830 *	14: 64,812,463	Mir124a-1hg	NA	NA	Mir124a-1hg
	rs3023460	17: 93,199,618	NA	NA	NA	NA
	rs3023517	19: 59,969,286	NA	NA	Fam204a	NA
Change	rs3023267	11: 65,710,376	NA	Dnah9	NA	Dnah9
	rs3660830 *	14: 64,812,463	Mir124a-1hg	NA	NA	Mir124a-1hg

The table provides information on chromosomal localization and regulation by phenotype selection (genetic effect), training (exercise), and phenotype selection with training. Font color of the gene symbol indicates lower expression (green) and higher expression (red) with respect to the control for each comparison (unselected or untrained). Underlined genes are considered as affected only by genetic effect because of no change of expression (log_2_FC) between phenotype selection and phenotype selection with training comparison. QTL regions with significant effect are marked with an *. Abbreviation: sed. = sedentary, tr. = trained, vs. = versus, NA = not available, SNP = single nucleotide polymorphism, bp = base pairs.

## Data Availability

Data are contained within the article or Supplementary Material.

## Supplementary Materials

The following are available online at https://www.mdpi.com/article/10.3390/cells10040736/s1, Table S1: List of significant (FDR ≤ 0.05) DEGs found in each group by comparing the corresponding chromosome, log_2_FC, and FDR. The group comparisons are reported in the following sequence: (**a**) DUC tr. vs. sed., (**b**) DUhTP tr. vs. sed., (**c**) DUhTP vs. DUC sed., (**d**) DUhTP vs. DUC tr. Table S2: List of DEGs and housekeeping genes selected for RT-qPCR validation with corresponding forward and reverse primers. Table S3: Comparison of log_2_FC of mRNA abundance measured with RNA-sequencing (NGS) and quantitative real-time PCR for selected genes; significant *p*-value in bold. Abbreviation: sed. = sedentary, tr. = trained, vs. = versus.
