# Supplementary material for: Control of Protein and Energy Metabolism in the Pituitary Gland in Response to Three-Week Running Training in Adult Male Mice
Source: Cells. 2021 Mar 26;10(4):736. doi: 10.3390/cells10040736 (PMC8065971; doi:10.3390/cells10040736)
Supplement: Supplementary file 1 [file cells-10-00736-s001.zip › cells-1131142 - supplementary_24032021.docx]

Supplementary Material

**Table S1.** Data sets of DEGs in the four comparison groups including the gene identification (Gen-ID), chromosome localization, fold change (log_2_FC), and false discovery rate (FDR) (a) DUC tr. vs. sed.; (b) DUhTP tr. vs. sed.; (c) DUhTP sed. vs DUC sed.; (d) DUhTP tr. vs. DUC tr. Abbreviation: DEGs = different expressed genes, sed. = sedentary, tr. = trained, vs. = versus.

**Table S2.** Primer sequences for quantitative real-time PCR for selected genes.

| **Gene** | **forward primer 5’→3’** | **reverse primer 5’→3’** |
| --- | --- | --- |
| Cdh22 | TACGTGGGCAAGATCCACTC | GTTTTCTGCTCCCGGTCCA |
| Chia1 | GGAATTGGTGCCCCTACCTC | GTCATAGCCAAGCCACTCGT |
| Col3a1 | AAGGCTGCAAGATGGATGCT | GGAGGGCCATAGCTGAACTG |
| Gabra3 | CTCTCTGCTTCGGGGAAGTG | CTTGGCTAGTGGTTCCAGGG |
| Lepr | TGAGCAGGCGTGCCATC | GTACCCGTCAGTTTCACATGATATATTG |
| Pdyn | CGACAGGGGGAGACTCTCAT | GGTGGCCGATCCAAGATTCA |
| Ppp1r17 | TAGTAGCAGGGCAGTCCGAT | TGAGGGTCTTCAGAGGAGAGG |
| Slc13a3 | TCCCACAGTGTGATGTGGTG | TCACCGCTTTAGCCTGATCC |
| Slc13a4 | CTAAGCCTTGGGCCGAGAAA | AGAGAGAGCAGCTGTAGGCT |
| Slc22a8 | TCCTGGTGGGTACCAGAGTC | AACCAGGCCAGAGAGAGACA |
| Slc7a11 | CTCCGAGGAGCAAGAGGAGTA | GATCACTGTTCGGTCGTGACT |
| Tnfrsf11b | AGGGCATACTTCCTGTTGCC | TGTTCATTGTGGTCCTCGGG |
| Vtn | CCTAAATCCTCGGACGGACG | GGGTCCCTTGATCAGTGGTG |
| Pgk1 | CAGTCTAGAGCTCCTGGAAGGT | GCCACTAGCTGAATCTTGCG |
| Rplp2 | GACGATGATCGGCTCAACAAG | ACCCTGAGCGATGACATCCT |
| Hprt | TCCTCCTCAGACCGCTTTT | CCTGGTTCATCATCGCTAATC |

Abbreviations: Cdh22 - Cadherin 22, Chia1 - chitinase, Col3a1 - collagen type III alpha 1, Gabra3 - gamma-aminobutyric acid A receptor subunit alpha 3, Lepr - leptin receptor, Pdyn - prodynorphin, Ppp1r17 - protein phosphatase 1 regulatory subunit 17, Slc13a3 - solute carrier family 13 member 3, Slc13a4 - solute carrier family 13 member 4, Slc22a8 - solute carrier family 22 member 8,Slc7a11 - solute carrier family 7 member 11, Tnfrsf11b - tumor necrosis factor receptor superfamily member 11b, Vtn - vitronectin, Pgk1 - phosphoglycerate kinase 1, Rplp2 - ribosomal protein, large P2, Hprt - hypoxanthine guanine phosphoribosyl transferase. The housekeeping genes are underlined.

**Table S3.** Comparison of log_2_FC of mRNA abundance measured with RNA-sequencing (NGS) and quantitative real-time PCR for selected genes; significant p-value in bold.

|  |  | **DUhTP sed. vs.**  **DUC sed.** | | **DUhTP tr. vs.**  **DUC tr.** | | **DUC**  **tr. vs. sed.** | | **DUhTP**  **tr. vs. sed.** | |
| --- | --- | --- | --- | --- | --- | --- | --- | --- | --- |
|  |  | **log_2_FC** | **p-value** | log_2_FC | p-value | log_2_FC | p-value | log_2_FC | p-value |
| Cdh22 | RNA seq | **1.3760** | **0.0002** | -0.1791 | 0.6110 | **1.5801** | **0.0000** | 0.0251 | 0.9437 |
|  | RT-qPCR | **1.2902** | **0.0003** | -0.0596 | 0.4452 | **1.3731** | **0.0252** | 0.0233 | 0.4539 |
| Chia1 | RNA seq | **1.3348** | **0.0000** | **0.5444** | **0.0168** | **0.7457** | **0.0014** | -0.0448 | 0.8426 |
|  | RT-qPCR | **1.2079** | **0.0000** | **0.6609** | **0.0097** | 0.5491 | 0.0882 | 0.0021 | 0.4918 |
| Col3a1 | RNA seq | **-0.7074** | **0.0000** | **-0.3648** | **0.0096** | **-0.5564** | **0.0001** | -0.2137 | 0.1303 |
|  | RT-qPCR | **-0.4558** | **0.0095** | -0.2152 | 0.1222 | **-0.4378** | **0.0320** | **-0.1972** | **0.0292** |
| Gabra3 | RNA seq | **1.4487** | **0.0003** | 0.2594 | 0.5017 | **1.2768** | **0.0013** | 0.0876 | 0.8206 |
|  | RT-qPCR | **1.5803** | **0.0000** | 0.0101 | 0.4937 | 1.6308 | 0.0727 | 0.0607 | 0.3648 |
| Lepr | RNA seq | **-1.3298** | **0.0000** | -0.0253 | 0.9329 | **-0.9968** | **0.0008** | 0.3076 | 0.3135 |
|  | RT-qPCR | **-1.1106** | **0.0372** | -0.2068 | 0.1605 | -0.7722 | 0.0994 | 0.1317 | 0.1224 |
| Pdyn | RNA seq | **2.2252** | **0.0000** | **1.3470** | **0.0000** | **0.8178** | **0.0014** | -0.0604 | 0.8088 |
|  | RT-qPCR | **2.2942** | **0.0004** | **1.7475** | **0.0002** | **0.5201** | **0.0155** | -0.0266 | 0.4691 |
| Ppp1r17 | RNA seq | **2.2716** | **0.0000** | 0.2429 | 0.5953 | **2.1606** | **0.0000** | 0.1319 | 0.7732 |
|  | RT-qPCR | **2.2930** | **0.0010** | 0.3059 | 0.2562 | **2.1149** | **0.0013** | 0.1278 | 0.3857 |
| Slc13a3 | RNA seq | **-1.9930** | **0.0013** | 0.1055 | 0.8604 | **-1.9446** | **0.0016** | 0.1538 | 0.7978 |
|  | RT-qPCR | -1.6433 | 0.0949 | -0.0047 | 0.4957 | -1.4154 | 0.1314 | 0.2231 | 0.2631 |
| Slc13a4 | RNA seq | **-3.8806** | **0.0000** | 0.1246 | 0.8662 | **-3.0815** | **0.0001** | 0.9237 | 0.2172 |
|  | RT-qPCR | -2.0278 | 0.0552 | 0.0233 | 0.4700 | -1.7479 | 0.0825 | 0.3032 | 0.1170 |
| Slc22a8 | RNA seq | **-3.7228** | **0.0000** | -0.3795 | 0.5686 | **-2.8533** | **0.0001** | 0.4900 | 0.4648 |
|  | RT-qPCR | **-1.5480** | **0.0489** | -0.4611 | 0.2696 | -0.4844 | 0.0800 | 0.6025 | 0.1945 |
| Slc7a11 | RNA seq | **-2.8264** | **0.0000** | -0.2677 | 0.6643 | **-2.4026** | **0.0002** | 0.1560 | 0.8014 |
|  | RT-qPCR | -2.9056 | 0.0556 | -0.6624 | 0.1463 | -1.8890 | 0.1040 | 0.3543 | 0.2737 |
| Tnfrsf11b | RNA seq | **-1.7033** | **0.0000** | 0.1376 | 0.6697 | **-1.3219** | **0.0000** | 0.5190 | 0.1209 |
|  | RT-qPCR | **-0.6450** | **0.0132** | **-0.3537** | **0.0306** | **-0.4697** | **0.0359** | -0.1784 | 0.2201 |
| Vtn | RNA seq | **-1.8826** | **0.0000** | -0.6484 | 0.0708 | **-1.2744** | **0.0004** | -0.0402 | 0.9107 |
|  | RT-qPCR | **-1.6723** | **0.0334** | **-0.4280** | **0.0177** | -1.1398 | 0.0833 | 0.1045 | 0.2262 |

Abbreviations: sed. = sedentary, tr. = trained, vs. = versus, Cdh22 - Cadherin 22, Chia1 - chitinase, Col3a1 - collagen type III alpha 1, Gabra3 - gamma-aminobutyric acid A receptor subunit alpha 3, Lepr - leptin receptor, Pdyn - prodynorphin, Ppp1r17 - protein phosphatase 1 regulatory subunit 17, Slc13a3 - solute carrier family 13 member 3, Slc13a4 - solute carrier family 13 member 4, Slc22a8 - solute carrier family 22 member 8,Slc7a11 - solute carrier family 7 member 11, Tnfrsf11b - tumor necrosis factor receptor superfamily member 11b, Vtn – vitronectin.
